# Identification of the Ghrelin and Cannabinoid CB_2_ Receptor Heteromer Functionality and Marked Upregulation in Striatal Neurons from Offspring of Mice under a High-Fat Diet

**DOI:** 10.3390/ijms22168928

**Published:** 2021-08-19

**Authors:** Jaume Lillo, Alejandro Lillo, David A. Zafra, Cristina Miralpeix, Rafael Rivas-Santisteban, Núria Casals, Gemma Navarro, Rafael Franco

**Affiliations:** 1 Centro de Investigación Biomédica en Red Enfermedades Neurodegenerativas (CiberNed), National Institute of Health Carlos III, Valderrebollo, 5, 28031 Madrid, Spain; lillojaume@gmail.com (J.L.); rrivasbioq@gmail.com (R.R.-S.); 2 Department of Biochemistry and Molecular Biomedicine, Universitat de Barcelona, 08028 Barcelona, Spain; 3 Department of Biochemistry and Physiology, Faculty of Pharmacy and Food Science, University of Barcelona, 08028 Barcelona, Spain; alilloma55@gmail.com (A.L.); dzafrasa7@alumnes.ub.edu (D.A.Z.); 4 Basic Sciences Department, Faculty of Medicine and Health Sciences, Universitat Internacional de Catalunya, 08190 Sant Cugat del Vallès, Spain; cristina.miralpeix@inserm.fr (C.M.); ncasals@uic.es (N.C.); 5 Centro de Investigación Biomédica en Red de Fisiopatología de la Obesidad y la Nutrición (CIBEROBN), Instituto de Salud Carlos III, Monforte de Lemos, 3, 28029 Madrid, Spain; 6 Institut de Neurociències, Universitat de Barcelona (UBNeuro), 08035 Barcelona, Spain; 7 School of Chemistry, University of Barcelona, 08028 Barcelona, Spain

**Keywords:** orexigenic, anorexia, marihuana, phytocannabinoids, G protein-coupled receptors, pharmacology, receptor heteromers, signaling, high-fat diet, obesity

## Abstract

Cannabinoids have been reported as orexigenic, i.e., as promoting food intake that, among others, is controlled by the so-called “hunger” hormone, ghrelin. The aim of this paper was to look for functional and/or molecular interactions between ghrelin GHSR1a and cannabinoid CB_2_ receptors at the central nervous system (CNS) level. In a heterologous system we identified CB_2_-GHSR1a receptor complexes with a particular heteromer print consisting of impairment of CB_2_ receptor/G_i_-mediated signaling. The blockade was due to allosteric interactions within the heteromeric complex as it was reverted by antagonists of the GHSR1a receptor. Cannabinoids acting on the CB_2_ receptor did not affect cytosolic increases of calcium ions induced by ghrelin acting on the GHSR1a receptor. In situ proximity ligation imaging assays confirmed the expression of CB_2_-GHSR1a receptor complexes in both heterologous cells and primary striatal neurons. We tested heteromer expression in neurons from offspring of high-fat-diet mouse mothers as they have more risk to be obese. Interestingly, there was a marked upregulation of those complexes in striatal neurons from siblings of pregnant female mice under a high-fat diet.

## 1. Introduction

The psychotropic effects of marihuana smoking led to the discovery of cannabinoid receptors and of endocannabinoids. Since the discovery of tetrahydrocannabinol (THC) and cannabidiol (CBD) in *Cannabis sativa* L., decades of research have led to the identification of multiple phytocannabinoids that show biological activity. The characterization of cannabinoid receptors, the use of animal models of disease, and the experience of marihuana users have led to the discovery of the health-promoting benefits of natural cannabinoids. In addition to the already approved cannabinoid-containing drugs (Dronabinol/Marinol^®^ -LGM Pharma Boca Raton, USA)- and Sativex^®^ and Epidiolex^®^ -Jazz Pharmaceuticals, Dublin, Ireland-), non-psychotropic compounds such as cannabidiol and cannabigerol extracted from *Cannabis sativa* L. have been proposed to aid in a variety of diseases (see [1,2] for review). Although cannabinoids can act via a variety of targets (GPR55, GPCR18, peroxisome proliferator-activated receptor gamma, serotonin receptors, etc.), their canonical targets are the cannabinoid CB_1_ and CB_2_ receptors. Both belong to the superfamily of G-protein-coupled-receptors (GPCRs) and both couple to the G_i_ heterotrimeric protein, i.e., receptor activation leads to G_i_-mediated deactivation of adenylyl cyclase and reduction of intracellular cAMP levels. The CB_1_ receptor (CB_1_R), expressed mainly in neurons, is the most abundant GPCR in the nervous system; while the CB_2_R is expressed at lower levels both in glia and neurons located in specific regions of the brain [3,4,5,6,7]. The CB_2_R is considered a target for neuroprotection, especially in diseases coursing with neuroinflammation. For instance, cannabinoids acting on the CB_2_R in striatal neural cells reduce the neuronal loss in synucleinopathies such as Parkinson’s disease [8,9,10,11,12,13]. The underlying mechanism is mainly related to the reduction of inflammation, but the CB_2_R expressed in some striatal neurons may also have a relevant role [3,7,14,15,16]. A selective antagonist of the CB_1_R, rimonabant, approved to combat obesity was, unfortunately, withdrawn due to side effects [17,18,19,20]. The anti-obesity potential of targeting the CB_2_R has not yet been addressed. 

Although the molecular mechanism has not been fully elucidated, the orexigenic properties of marihuana consumption are well known [21]. Ghrelin is often considered the “hunger” hormone because its appearance in blood leads to an increase under food intake [22,23]. Ghrelin, a 28-amino acid peptide, mainly produced by specialized cells of the gastrointestinal act, may reach the central nervous system where it activates central mechanisms that control food intake [24,25,26,27]. Its main target there is the ghrelin GHSR1a receptor, also a member of the GPCR superfamily [28]. The aim of this paper was to investigate the molecular and/or functional interactions between CB_2_ and GHS1a receptors that could explain the orexigenic effects of marihuana consumption and whether ghrelin affects the neuroprotective effects induced by cannabinoids acting on the CB_2_R. As offspring from obese mothers have more risk to be obese, the potential expression of complexes formed by these two receptors was also studied in neurons isolated from fetuses of pregnant female mice in a high-fat diet.

## 2. Results

### 2.1. The CB_2_R May Interact with the GHSR1a

**Figure 1:**
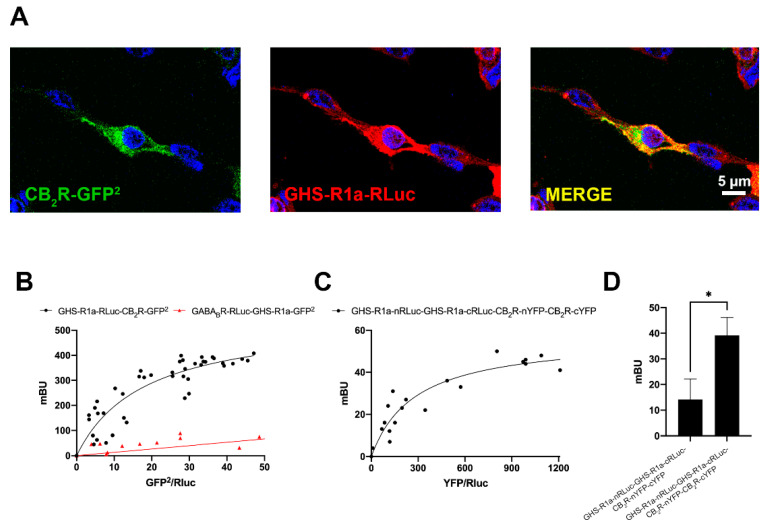
Molecular interaction between GHS1a and CB_2_ receptors expressed in transfected HEK-293T cells. (**A**) Confocal microscopy images of HEK-293T cells co-transfected with cDNAs for GHSR1a-RLuc (0.75 µg) and for CB_2_R-GFP^2^ (0.5 µg). GHSR1a-RLuc (red) was identified by immunocytochemistry using an anti-RLuc antibody (Merck-Millipore, 1/100). The CB_2_R-GFP^2^ (green) was identified by the fluorescence due to GFP^2^. colocalization is shown in yellow (image in the right). Cell nuclei were stained with Hoechst (blue). Scale bar: 5 μm. (**B**) BRET saturation experiments were performed using HEK-293T cells co-transfected with (i) a constant amount of GHSR1a-RLuc cDNA (1.5 μg) and increasing amounts of CB_2_R-GFP^2^ cDNA (0–2 μg) or (ii) a constant amount of GABA_B_-RLuc cDNA (0.75 μg) and increasing amounts of GHSR1a-GFP^2^ cDNA (0–2 μg) as negative control. BRET data are expressed as the mean ± SEM of 8 different experiments performed in duplicates. (**C**) Bimolecular luminescence and fluorescence complementation (BiLFC) assays were performed in HEK-293T cells co-transfected with a constant amount of GHSR1a-cRLuc and GHSR1a-nRLuc cDNAs (1.5 μg each) and increasing amounts of CB_2_R-cYFP and CB_2_R-nYFP cDNAs (0–3 μg each). (**D**) HEK-293T cells were co-transfected with 1.5 μg of the GHSR1a-cRLuc and GHSR1a-nRLuc cDNAs and 3 μg of the CB_2_R-nYFP and CB_2_R-cYFP cDNAs or with the non-fused cYFP as negative control. BRET data are expressed as the mean ± SEM of 7 different experiments performed in duplicates. * *p* < 0.05. mBU: milliBret units.

Consumption of *Cannabis sativa* L. increases appetite via a mechanism in which ghrelin, a hormone of the endocrine system, participates. To investigate whether there may be a functional interaction between the cannabinoidergic and the orexinergic systems, we first investigated a potential interaction between the cannabinoid receptor CB_2_ and the functional form of the ghrelin receptor, GHSR1a. Immunocytochemical assays in HEK-293T cells transfected with the cDNA for the CB_2_R fused to the Green Fluorescent Protein (GFP^2^) and the cDNA of the GHSR1a fused to Renilla luciferase (RLuc) led to detect the receptors at the plasma membrane level with a marked colocalization (Figure 1A). As colocalization may be found for proteins that are close (approximately 200 nm apart) but not directly interacting, a Bioluminescence Resonance Energy Transfer (BRET) assay was performed in HEK-293T cells cotransfected with a constant amount of the cDNA for GHSR1a-RLuc and increasing amounts of cDNA for CB_2_R-GFP^2^. A saturation curve (BRET_max_: 550 ± 50 mBU, BRET_50_: 18 ± 4) was obtained, demonstrating a direct interaction between the two receptors in the heterologous expression system (Figure 1B). As negative control, HEK-293T cells were transfected with a constant amount of GABA_B_R-RLuc cDNA and increasing amounts of GHSR1a-GFP^2^ cDNA; the nonspecific linear signal indicated a lack of interaction between these two proteins.

It is well known that GPCRs may form homodimers of heterodimers and higher order structures. One of the first examples has been provided for the adenosine A_1_ and A_2_A receptors that can form tetrameric structures complexed with two heterotrimeric G proteins, one G_i_ and one G_s_ [29,30]. To investigate the possible formation of tetramers formed by CB_2_ and GHS1a receptors, BRET with bimolecular complementation experiments was performed in HEK-293T cells transfected with a constant amount of Rluc8 N/C hemiproteins fused to GHSR1a (GHSR1a-cRLuc and GHSR1a-nRLuc) and increasing amounts of YFP N/C hemiproteins fused to CB_2_R (CB_2_R-cYFP and CB_2_R-nYFP). As observed in Figure 1C, both CB_2_R and GHSR1a were able to reconstitute RLuc and YFP proteins, emitting, respectively, bioluminescence and fluorescence. This result proves homodimerization. However, a saturation BRET curve was possible in cells expressing a constant amount of donor complemented hemiproteins and increasing levels of acceptor complemented hemiproteins (BRET_max_: 56 ± 5 mBU, BRET_50_: 280 ± 70 mBU). Taken together, these results constitute evidence of heterotetramer formation with two protomers of each receptor, i.e., a heterotetramer constituted by two homodimers. As a negative control, we obtained a negligible signal when substituting one of the fused proteins by the non-fused version (one example is provided in Figure 1D that shows data in assays where CB_2_R-cYFP cDNA was substituted by that of cYFP). 

### 2.2. CB_2_R-Mediated Signaling Is Blocked in the CB_2_-GHSR1a Receptor Heteromer (CB_2_-GHSR1aHet)

**Figure 2:**
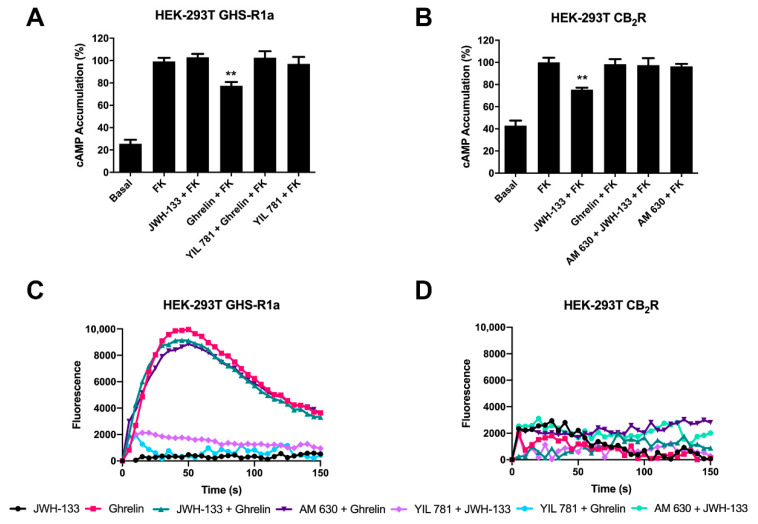
Functional characterization of GHS1a and CB_2_ receptors expressed in HEK-293T cells. (**A**,**B**) HEK-293T cells transfected with plasmids encoding for either GHSR1a (1.5 μg) (**A**) or CB_2_R (1 μg) (**B**) were pre-treated with selective antagonists, 1 μM YIL 781-GHSR1a- or 1 μM AM 630-CB_2_R, and subsequently treated with the selective agonists, 200 nM ghrelin -GHSR1a- or 200 nM JWH-133 -CB_2_R-. cAMP levels after 0.5 μM forskolin stimulation were detected by the Lance Ultra cAMP kit and the results were expressed in % respect to levels obtained upon forskolin stimulation. The values are the mean ± SEM of 10 different experiments performed in triplicates. One-way ANOVA followed by Dunnett’s multiple comparison post-hoc test were used for statistical analysis. ** *p* < 0.01, versus forskolin treatment. (**C**,**D**) HEK-293T cells expressing an engineered calcium sensor, GCaMP6 and GHSR1a (**C**) or CB_2_R (**D**) were pre-treated with selective antagonists for 10 min followed by agonist stimulation. Representative traces of intracellular Ca^2+^ responses over time are shown (6 independent experiments).

**Figure 3:**
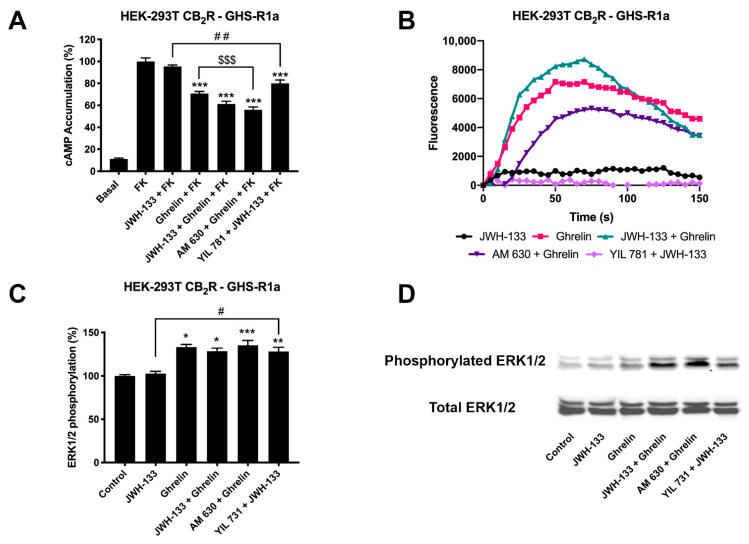
CB_2_-GHSR1aHet-mediated signaling in transfected HEK-293T cells. Panels (**A**–**C**) HEK-293T cells were transfected with cDNAs encoding for GHSR1a (1.5 μg) and CB_2_R (1 μg) (**A,C**) or with GHSR1a (1.5 μg), CB_2_R (1 μg) and the GCaMP6 calcium sensor (**B**) and pre-treated with antagonists, 1 μM YIL 781 -for GHSR1a- and 1 μM AM 630 -for CB_2_R, and subsequently stimulated with selective agonists, 200 nM ghrelin -for GHSR1a- and 200 nM JWH-133 -for CB_2_R, individually or in combination. cAMP levels were analyzed by the Lance Ultra cAMP kit and results were expressed in % respect to levels obtained upon 0.5 μM forskolin stimulation (**A**). Representative traces of intracellular Ca^2+^ responses over time are shown (9 independent experiments) (**B**). ERK 1/2 phosphorylation was determined by immunoblot using the Odyssey infrared scanner (LI-COR Biosciences) (**C,D**). In cAMP accumulation and MAPK pathway signaling-related assays, the values are the mean ± SEM of 8 different experiments performed in triplicates. One-way ANOVA followed by Dunnett’s multiple comparison post-hoc test were used for statistical analysis..* *p* < 0.05, ** *p* < 0.01, versus basal in pERK1/2 assays; *** *p* < 0.001; versus forskolin treatment in cAMP or versus basal in pERK1/2 assays, # *p* < 0.05 versus JWH-133 treatment in pERK assays, ## *p* < 0.01 versus JWH-133+FK treatment in cAMP assays; $$$ *p <* 0.001; versus ghrelin+FK treatment.

Once a direct interaction between CB_2_R and GHSR1a was identified, we investigated the functional consequences of interprotomer cross-talk within the heteromer. Signaling assays were performed taking into account that both CB_2_ and GHS1a receptors may couple to G_i_. The activation of the receptors leads to reduced adenylate cyclase activity and a decrease in cytosolic cAMP levels. Accordingly, intracellular cAMP levels following receptor activation were measured in HEK-293T cells expressing CB_2_R, GHSR1a or both. In cells expressing the GHSR1a, ghrelin induced a 25% decrease of forskolin-induced cAMP levels that was completely counteracted by YIL 781 (1 µM), the selective GHSR1a antagonist. Moreover, the selective CB_2_R agonist, JWH-133 induced no effect, demonstrating the ligand selectivity and lack of nonspecific effects (Figure 2A). On the other hand, in cells expressing the CB_2_R, 200 nM JWH-133 induced a 30% decrease with respect to forskolin-induced cAMP levels, an effect that was completely blocked by pretreatment with the selective antagonist (AM 630, 1 µM). The control that was performed with 200 nM ghrelin induced no effect (Figure 2B). In HEK-293T cells expressing both receptors, the effect of ghrelin was similar to that found in cells only expressing the ghrelin receptor (Figure 3A); remarkably, the selective CB_2_R agonist, JWH-133, did not induced any effect suggesting that CB_2_R, in the heteromeric context, is not functionally coupled to G_i_. Simultaneous treatment with both agonists resulted in a 37% decrease of the intracellular cAMP levels, i.e., similar to levels obtained upon ghrelin treatment. However, when the same cells were pretreated with ghrelin selective antagonist (YIL 781, 1µM) JWH-133 was able to allow the CB_2_R-G_i_ coupling (Figure 3A). Additionally, the finding that 1 µM AM 630 in co-transfected HEK-293T cells potentiated GHSR1a-mediated signaling was also noticeable. These results indicate that (i) the basal structure restrains activation and/or decreases the functional signal, and (ii) the CB_2_R blockade disappears in the presence of the selective antagonists of the partner receptor in the macromolecular complex.

We next analyzed the possibility of calcium mobilization upon activation of receptors. It is well known that GHSR1a receptor may couple to G_q_, thus being linked to phospholipase C activation and calcium release from endoplasmic reticulum stores towards the cytosol. In GHSR1a-expressing HEK-293T cells, 200 nM ghrelin led to calcium mobilization (Figure 2B), that was counteracted by the pretreatment of the GHSR1a antagonist but not by the CB_2_R antagonist (and did not occur in cells only expressing the CB_2_R). In GHSR1a- or in CB_2_-receptor-expressing cells, JWH-133 treatment did not lead to any effect (Figure 2D). 

When calcium mobilization was assayed in cotransfected cells, ghrelin treatment resulted in a characteristic curve of calcium mobilization that was not significantly modified upon simultaneous treatment with JWH-133 (Figure 3B). Pretreatment with the CB_2_R antagonist, AM 630, partially blocked the ghrelin effect. This phenomenon, known as cross-antagonism is, often, a print of the heteromer that is instrumental to detect it in natural sources. In these cells the CB_2_R was not coupled to G_q_, i.e., JWH-133 did not lead to any signal related to changes in cytosolic levels of the calcium ion.

Finally, as CB_2_R and GHSR1a activation leads to activation of the mitogen-activated protein kinase (MAPK) pathway [31,32], we tested the properties of the heteromer in the link to the MAPK signaling cascade. Hence, we measured ERK1/2 phosphorylation in HEK-293T cells expressing the two receptors. Treatment with 200 nM ghrelin resulted in a significant signal whereas 200 nM JWH-133 did not induce any effect (Figure 3C). Interestingly, pretreatment with the ghrelin receptor antagonist, YIL 781, allowed the activation of the MAPK pathway via the CB_2_R. This action that results from antagonizing the GHSR1a was similar to that found in cAMP assays, i.e., blockade of the ghrelin receptor releases the brake posed on CB_2_R-mediated signaling.

### 2.3. CB_2_R Activation Is Blocked in Striatal Neurons via Formation of Heteromers of CB_2_R and GHSR1a (CB_2_R-GHSR1aHets)

Upon the demonstration that CB_2_R-GHSR1aHets may be formed in a heterologous system and upon the detection of particular heteromer prints, we undertook the search for detecting the prints in a more physiological context; for such purpose we used primary striatal neurons isolated from fetuses of mothers under a standard (STD) diet (see Section 4, Materials and Methods). Primary striatal neurons from fetuses of pregnant female mothers were isolated and cultured for 15 days prior to undertake signaling assays.

**Figure 4:**
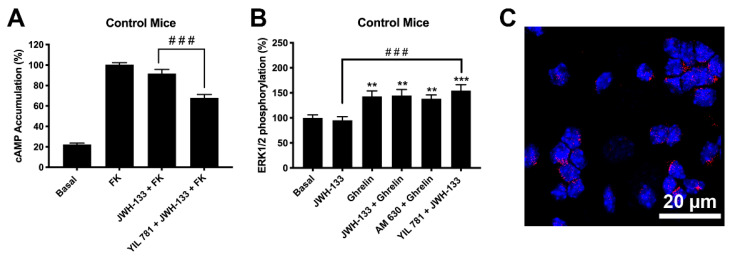
Expression and function of CB_2_R-GHSR1aHets in primary neurons from C57BL/6J mice. (**A**,**B**) Primary striatal neurons isolated from C57BL/6J mice were pre-treated with antagonists, 1 μM YIL 781 -for GHSR1a- or 1 μM AM 630 -for CB_2_R-, and subsequently stimulated with selective agonists, 200 nM ghrelin -for GHSR1a- or 200 nM JWH-133 -for CB_2_R-, individually or in combination. cAMP levels were determined using the Lance Ultra cAMP kit and results were expressed in % respect to levels obtained upon 0.5 μM forskolin stimulation (**A**), while ERK1/2 phosphorylation was analyzed using the AlphaScreen^®^SureFire^®^ kit (PerkinElmer; Wellesley, MA, USA) (**B**). Values are the mean ± SEM of 6 different experiments performed in triplicates. One-way ANOVA followed by Dunnett’s multiple comparison post-hoc tests were used for statistical analysis. ** *p*<0.01, *** *p* < 0.001 versus basal, ### *p <* 0.001; versus JWH-133+FK treatment in cAMP or versus JWH-133 treatment in p-ERK1/2 assays. (**C**) CB_2_R-GHSR1aHets were detected in primary striatal neurons by the in situ proximity ligation assay (PLA) using specific antibodies. Cell nuclei was stained with Hoechst (blue). Samples from 5 different animals were processed and analyzed and quantitation is shown in Figure 5. Scale bar: 20 μm.

First, in regard to the forskolin-induced levels, the cAMP assays showed that stimulation of CB_2_R with JWH-133 did not induce any significant effect (Figure 4A). These findings may be due to lack of CB_2_R or to the presence of CB_2_R-GHSR1aHets. However, the results using antagonists did show that the CB_2_R is expressed and that is likely forming complexes with the ghrelin receptor. In fact, YIL 781 allowed the G_i_ coupling of the CB_2_R as JWH-133 was then able to decrease the forskolin-induced cytosolic cAMP levels (Figure 4A). These results are fully consistent with those obtained in the heterologous expression system (HEK-293T cells). In addition, analysis of the link to the MAPK pathway showed the effect of ghrelin whereas the effect of JWH-133 was only possible in cells pretreated with the selective ghrelin receptor antagonist (Figure 4B). 

Finally, we used an imaging technique, the Proximity Ligation Assay (PLA), to demonstrate the occurrence of CB_2_R-GHSR1aHets in striatal neurons. PLA has been instrumental for detecting, in natural scenarios (cells or tissues), complexes formed by two proteins. Clusters of macromolecules formed by two different proteins appear as red dots using a confocal microscope (see Section 4, Materials and Methods for details); such red dots, which surrounded Hoechst stained nuclei, demonstrated the existence in striatal neurons of complexes of CB_2_R and GHSR1a (Figure 4C).

### 2.4. Expression of CB_2_R and GHSR1a Complexes Is Increased in Neurons of Progeny from Mothers on a High-Fat Diet

The ghrelin GHS1a receptor has an important role in controlling food intake, with some authors referring to ghrelin as the peripheral “hunger hormone” [22]. Moreover, it is known both an association between child obesity and maternal body mass index [33] and that diet-induced obesity leads to neuroinflammation and synapsis structure modification [34]. Herein, we investigated the expression of the CB_2_R-GHSR1aHet in striatal neurons of the progeny from female mice under a high-fat (HFD) diet. Primary striatal neurons from fetuses of pregnant HFD mothers were isolated and cultured for 15 days prior to undertaking signaling assays, and the results were compared with those obtained from neurons isolated from fetuses of mothers under a standard diet (see results presented in the previous section). 

**Figure 5:**
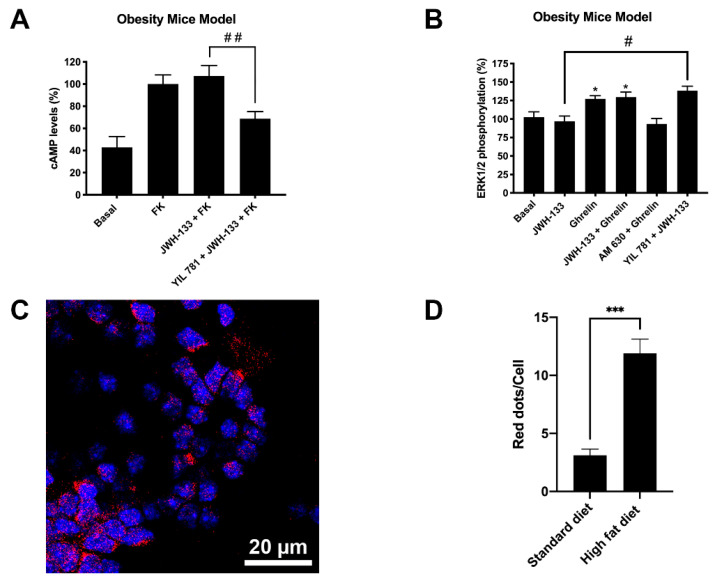
Expression and function of CB_2_R-GHSR1aHets in primary neurons isolated from fetuses of pregnant C57BL/6J female mice subjected to a high-fat diet. (**A**,**B**) Primary striatal neurons isolated from fetuses of pregnant females mice subjected to a high-fat diet for 60 days were pre-treated with antagonists, 1 μM YIL 781 -for GHSR1a- or 1 μM AM 630 -for CB_2_R-, followed by stimulation with selective agonists, 200 nM ghrelin -for GHSR1a- or 200 nM JWH-133 -for CB_2_R-, individually or in combination. cAMP levels were determined using the Lance Ultra cAMP kit and results were expressed in % versus 0.5 µM forskolin treatment (**A**) while ERK1/2 phosphorylation was analyzed using the AlphaScreen^®^ SureFire^®^ kit (PerkinElmer) (**B**). Values are the mean ± SEM of 5 different experiments performed in triplicates. One-way ANOVA followed by Dunnett’s multiple comparison post-hoc test were used for statistical analysis. # *p <* 0.05, ## *p <* 0.005 versus JWH-133+FK treatment in cAMP or versus JWH-133 treatment in p-ERK1/2 assays. * *p* < 0.05, basal. (**C**). CB_2_R-GHSR1aHets were detected by the in situ proximity ligation assay (PLA) in primary striatal neurons; the negative control undertaken by omitting one of the primary antibodies is shown in Supplementary Figure S1. Experiments were performed in samples from 5 animals. The number of red dots/cell was quantified using the Andy’s algorithm Fiji’s plug-in and represented over cell stained nuclei with Hoechst (blue) (**D**). The number of dots-clusters/cell were compared to those in neurons from control mice (mice fed with standard diet). Unpaired t-test was used for statistics analysis. *** *p* < 0.001, versus control. Scale bar: 20 μm.

By analyzing the cAMP levels in primary striatal neurons from mothers in a HFD (Figure 5A) the results were qualitatively similar to those observed in the control group (Figure 4A) although more marked, i.e., the decreases obtained with respect to forskolin-induced cAMP levels were higher. One print of the CB_2_R-GHSR1aHet was noticed as JWH-133 was only efficacious in the presence of the selective GHS1a receptor antagonist (YIL 781). The results related to the phosphorylation of pERK1/2 (Figure 5B) were virtually identical to those found in the samples of the control group (Figure 4B), being necessary YIL 781 to observe an effect of JWH-133.

Finally, when analyzing by PLA the CB_2_R-GHSR1aHet expression, a marked increase in samples from the HFD group was noticed. An average of 12 red dots per Hoechst stained nuclei were observed in neurons derived from the HFD group, whereas neurons derived from the standard diet group only presented three red dots per stained nuclei (Figure 4C and Figure 5C,D the negative control is provided in Supplementary Figure S1). Remarkably, the striatal neurons of the siblings of HFD mothers show a much higher number of CB_2_R-GHSR1aHets than neurons of the siblings of mothers in standard diet, suggesting an enhanced suppression of CB_2_R function in HFD mother’s siblings.

## 3. Discussion

There is interest in the potential of targeting cannabinoid receptors for combating a variety of diseases. Despite targeting the CB_1_R was the main objective in cannabinoid-related drug discovery, the psychotropic action of some cannabinoids acting on its receptors and the side effects of a CB_1_R antagonist approved to combat obesity, rimonabant, has shifted the focus toward the CB_2_R. The limited expression of the CB_2_R in some CNS regions and its upregulation in activated glial cells have led to propose this receptor as target to limit neuroinflammation, to limit neurotoxicity induced by oxidative stress, to afford neuroprotection and/or the increase neurogenesis/gliogenesis [11,35,36,37,38,39,40,41]. Medications targeting cannabinoid receptors have been approved for a very limited therapeutic interventions (Sativex^®^, Marinol^®^, Epidiolex^®^; mainly to combat spasticity and emesis). The potential is higher and there are clinical trials already running or in preparation to test the efficacy of cannabidiol, an allosteric modulator of the CB_2_R [42], and of other cannabinoids for treating from the hypoxia of the neonate to improving the course of amyotrophic lateral or multiple sclerosis [15,43,44,45,46,47,48,49,50,51]. The number of registered clinical trials indicated in https://clinicaltrials.gov/ for testing cannabidiol in a variety of pathological conditions is 321 (date: 1 July 2021). As commented in the introduction, the CB_2_R is, also, an attractive target to afford neuroprotection in Parkinson’s disease [3,7,8,9,10,11,12,13,14]. 

The ghrelin receptor expressed in the hypothalamus and in reward circuits of the brain is key to mediating the control of food intake [24,25,52,53,54]. Several of the known phytocannabinoids are able to enter the brain where they exert multiple actions [1,12,55,56,57,58,59,60,61,62]. This study was undertaken to identify possible interactions between the orexinergic and the cannabinoidergic systems. The discovery of complexes formed by GHS1a and CB_2_ receptors and their identification in striatal neurons show that the ghrelin receptor modulates the effect of cannabinoids in the brain. From the molecular point of view our results suggest a tetrameric structure in complex with, at least, one G_i_ and one G_q_ protein. These results are qualitatively similar to those reported in the first reliable structural model of two interacting GPCRs, namely A_1_ and A_2A_ adenosine receptors that arrange into a tetramer formed by two homodimers and are coupled to two different G proteins (one G_s_ and one G_i_) [29,30]. In this example, the activation of one receptor blocks the activation of the partner receptor in the heteromer. However, the allosteric interactions within the CB_2_R-GHSR1aHet are such that CB_2_R-mediated signaling is blocked even in the absence of ghrelin, i.e., irrespective of the presence of the hormone, the CB_2_R cannot be activated within the CB_2_R-GHSR1aHet. Cannabinoid receptor activation is only possible in the presence of a selective GHS1a receptor antagonist, YIL 781. Although this atypical behavior is not found in many of the already identified GPCR heteromers (http://www.gpcr-hetnet.com/, accessed on 24 June 2021; [63]), it has been reported that the mere presence of the A_2B_ receptor and the formation of A_2A_–A_2B_ receptor heteromers decreases both agonist affinity and function of the A_2A_ receptor. At present, the only reasonable hypothesis to explain the physiological role of the of CB_2_R-GHSR1aHet and of A_2A_R-A_2B_RHet is that they are formed to suppress the functionality of one of the two receptors in the heteromer.

Heteromer formation in GPCRs appear as a means for achieving functional diversification [64,65,66], i.e., heteromers are functional units that behave differently than individually-expressed receptors. There have been few ghrelin receptor-containing heteromers reported in the literature. To our knowledge, GHSR1a may interact with the class A dopamine D_1_ and D_2_ receptors or with class B secretin receptors [67,68]. It should be noted that cocaine interacting with sigma1-receptors modulate the GHSR1a-D_1_ receptor interaction in hypothalamic cells to suppress appetite [69]. Results in the present paper show that CB_2_R functionality is blunted by formation of the CB_2_R-GHSR1aHet. This discovery is complemented by another result of the present study, namely that the expression of the CB_2_R-GHSR1aHet in primary striatal neurons is altered in the progeny of obese mothers. The increased CB_2_R-GHSR1aHets expression in samples from fetuses of mothers subjected to a HFD (when compared with samples from fetuses of mothers subjected to a STD) may explain some of the findings related to obesity and unbalanced diets. On the one hand, genetic inactivation of the gene coding for the receptor leads to improved insulin function but leads to age-related obesity [70]. On the other hand, neuroinflammation often occurs in obesity [71] and, interestingly, balancing the diet improves both maternal deficits and neuroinflammation in offspring [72]. These findings are relevant as cumulative research has found a higher proportion of obesity cases among children with obese parents [73]. Upregulation of the CB_2_R-GHSR1aHet in offspring of mothers with HFD would indicate that already at birth these animals have CB_2_R function compromised, i.e., the benefits of cannabinoids acting on striatal CB_2_Rs would be lost by the blockade exerted by the ghrelin receptor. In addition, our results suggest that GHSR1a antagonists could have a double benefit: (i) reducing food intake and (ii) revert the detrimental effects of HFD on the functionality of striatal CB_2_Rs.

## 4. Materials and Methods

### 4.1. Reagents

JWH-133, AM 630, Ghrelin and YIL 781 were purchased from Tocris Bioscience (Bristol, UK). 

### 4.2. High Fat Diet Model Generation

C57BL/6J female mice were used for the experiments. All animals were housed on a 12 h/12 h light/ dark cycle in a temperature- and humidity-controlled room and were allowed free access to water and standard laboratory chow. C57BL/6J mice were randomly assigned to a high fat diet (HFD) (60% kcal from fat; catalog no. D12492, Research Diets, New Brunswick, NJ, USA) or standard diet (STD) (10% kcal from fat; catalog no. D12450B, Research Diets) for 60 days. Primary striatal neurons were obtained from fetuses of mother on STD or HFD. Pregnant animals were killed by cervical dislocation during the light phase. All animal procedures were performed in agreement with European guidelines (2010/63/EU) and approved by the University of Barcelona Ethical Committee, which reports to the regional Government (Protocol #9659; Generalitat de Catalunya, 24 May 2019).

### 4.3. Cell Culture and Transient Transfection

Human embryonic Kidney HEK-293T (lot 612968) cells were acquired from the American Type Culture Collection (ATCC, Manassas, VA, USA). They were amplified and frozen in liquid nitrogen in several aliquots. Cells from each aliquot were used until passage 19. HEK-293T cells were grown in Dulbecco’s modified Eagle’s medium (DMEM) (Gibco, Waltham, MA, USA) supplemented with 2 mM L-glutamine, 100 U/mL penicillin/streptomycin, MEM Non-Essential Amino Acid Solution (1/100) and 5% (*v*/*v*) heat-inactivated fetal bovine serum (FBS) (all supplements were from Invitrogen, Paisley, Scotland, UK) and maintained in a humid atmosphere of 5% CO_2_ at 37 °C.

Cells were transiently transfected with the corresponding cDNAs using the PEI (PolyEthylenImine, Sigma-Aldrich, St. Louis, MO, USA) method as previously described [74,75]. 4 h after transfection, growth medium was replaced by complete medium. Experiments were carried out 48 h later.

To prepare primary striatal neurons, brains from fetuses of pregnant C57/BL6 mice were removed (gestational age: 17 days). Neurons were isolated as described in Hradsky et al. [1] and plated at a confluence of 40,000 cells/0.32 cm^2^. Briefly, the samples were dissected and, after careful removal of the meninges, digested for 20 min at 37 °C with 0.25% trypsin. Trypsinization was stopped by adding an equal volume of culture medium (Dulbecco’s modified Eagle medium-F-12 nutrient mixture, Invitrogen). Cells were brought to a single cell suspension by repeated pipetting followed by passage through a 100 μm-pore mesh. Pelleted (7 min, 200× *g*) cells were resuspended in supplemented DMEM and seeded at a density of 3.5 × 10^5^ cells/mL in 6-well plates. The day after, medium was replaced by neurobasal medium supplemented with 2 mM L-glutamine, 100 U/mL penicillin/streptomycin and 2% (*v*/*v*) B27 medium (GIBCO). Neuronal cultures were used for assays after 15 days of culture. Using NeuN as a marker, the percentage of neurons in the cultures was >90%.

### 4.4. Expression Vectors

The human cDNAs for the CB_2_, GHS1a and GABA_B_ receptors cloned in pcDNA3.1 were amplified without their stop codons using sense and antisense primers. The primers harbored either unique BamHI and HindIII sites for GHS1a and GABA_B_ receptors and BamHI and KpnI sites for the CB_2_R. The fragments were subcloned to be in frame with an enhanced green fluorescent protein (GFP^2^-N2, Clontech, Heidelberg, Germany), the Renilla luciferase protein (RLuc) (pRluc-N1; PerkinElmer, Wellesley, MA, USA) or the hemiproteins nRLuc8, cRLuc8, nVenus or cVenus (pcDNA3.1-nRLuc8, pcDNA3.1-cRLuc8, pcDNA3.1-nVenus and pcDNA3.1-cVenus) on the C-terminal end of the receptor to produce CB_2_R-GFP^2^, GHSR1a-GFP^2^, GHSR1a-RLuc, GABA_B_R-RLuc, GHSR1a-nRLuc, GHSR1a-cRLuc, CB_2_R-nYFP and CB_2_R-cYFP fusion proteins.

### 4.5. Immunofluorescence 

HEK-293T cells transfected with cDNAs for CB_2_R-GFP^2^ and GHSR1a-RLuc were fixed in 4% paraformaldehyde for 15 min and then washed twice with PBS containing 20 mM glycine before permeabilization with the same buffer containing 0.2% Triton X-100 (5 min incubation). The samples were treated for 1 h with blocking solution (PBS containing 1% bovine serum albumin) and labeled with a mouse anti-RLuc (1/100; MAB4400, Millipore, Burlington, MA, USA) as primary antibody and a Cy3-conjugated anti-mouse IgG (1/200; 715-166-150; Jackson ImmunoResearch) as secondary antibody. The samples were washed several times and mounted with 30% Mowiol (Calbiochem, San Diego, CA, USA). Nuclei were stained with Hoechst (1/100). Samples were observed under a Zeiss 880 confocal microscope (Leica Microsystems, Wetzlar, Germany).

### 4.6. Bioluminescence Resonance Energy Transfer (BRET) Assay

HEK-293T cells growing in 6-well plates were transiently co-transfected with a constant amount of cDNA encoding for GHSR1a-Rluc and with increasing amounts of cDNA for CB_2_R-GFP^2^. For negative control, cells were co-transfected with a constant amount of cDNA encoding for GABA_B_R-Rluc and with increasing amounts of cDNA for GHSR1a-GFP^2^. 48 h post-transfection, cells were washed twice in quick succession with HBSS (137 mM NaCl; 5 mM KCl; 0.34 mM Na_2_HPO_4_; 0.44 mM KH_2_PO_4_; 1.26 mM CaCl_2_; 0.4 mM MgSO_4_; 0.5 mM MgCl_2_ and 10 mM HEPES, pH 7.4) supplemented with 0.1% glucose (*w*/*v*), detached by gently pipetting and resuspended in the same buffer. To have an estimation of the number of cells per plate, protein concentration was determined using a Bradford assay kit (Bio-Rad, Munich, Germany) using bovine serum albumin dilutions for standardization. To quantify GFP^2^-fluorescence expression, cells were distributed (20 μg protein) in 96-well microplates (black plates with a transparent bottom; Porvair, Leatherhead, UK). Fluorescence was read using a fluorimeter FluoStar Optima (BMG Labtechnologies, Offenburg, Germany) equipped with a high-energy xenon flash lamp, reading at 510 nm. GFP^2^-fluorescence expression was determined as the fluorescence of the sample minus the fluorescence of cells only expressing protein-RLuc. For the BRET^2^ measurements, the equivalent of 20 μg protein of cell suspension was distributed in 96-well microplates (white plates; Porvair), and 5 μM Deep-Blue C was added (PJK GMBH, Kleinblittersdorf, Germany). 30 s after, readings were collected using a Mithras LB 940 (Berthold, Bad Wildbad, Germany), which allowed the integration of the signals detected in the short-wavelength filter at 410 nm (400–420 nm) and the long-wavelength filter at 510 nm (500–520 nm). To quantify receptor-RLuc expression, luminescence readings were collected 10 min after 5 μM coelenterazine H addition. The net BRET is defined as [(long-wavelength emission)/(short-wavelength emission)]-Cf where Cf corresponds to [(long-wavelength emission)/(short-wavelength emission)] for the RLuc construct expressed alone in the same experiment. The BRET curves were fitted assuming a single phase by a non-linear regression equation using the GraphPad Prism software (San Diego, CA, USA). BRET values are given as milli BRET units (mBU: 1000 × net BRET).

### 4.7. BRET with Bimolecular Luminescence and Fluorescence Complementation (BiLFC)

For BRET with bimolecular luminescence and fluorescence complementation (BiLFC) assays, HEK-293T cells were transiently transfected with a constant amount of cDNAs for GHSR1a-cRLuc and for GHSR1a-nRLuc cDNAs and increasing amounts of cDNAs for CB_2_R-cYFP and for CB_2_R-nYFP cDNAs. For negative controls, the cDNA for one of the fusion proteins was substituted by the corresponding empty vector (pcDNA3.1-cYFP) maintaining the other three plasmids. Protein determination was performed as described in the previous section. 48 h post-transfection, the equivalent of 20 μg protein of cell suspension was distributed in 96-well microplates. To quantify protein-YFP expression, fluorescence was read in a Mithras LB 940 equipped with a high-energy xenon flash lamp, using a 30 nm bandwidth excitation filter at 485 nm and an emission filter at 530 nm (510–550 nm). For BRET measurements, readings were collected 1 min after the addition of 5 μM coelenterazine H (Molecular Probes, Eugene, OR, USA) using a Mithras LB 940, which allows the integration of the signals detected in the short-wavelength filter at 485 nm and the long-wavelength filter at 530 nm. To quantify protein-RLuc expression, luminescence readings were obtained 10 min after 5 μM coelenterazine H addition using a Mithras LB 940.

### 4.8. cAMP Determination

HEK-293T cells transfected with the cDNAs for CB_2_R (0.5 µg) and/or GHSR1a (1 µg) and neuronal primary cultures were plated in 6 well plates. Two hours before initiating the experiment, neuronal culture or HEK-293T cell-culture media were exchanged to non-supplemented DMEM medium. Then, cells were detached, re-suspended in non-supplemented medium containing 50 µM zardaverine, and plated in 384-well microplates (2500 cells/well). Cells were pretreated (15 min) with the corresponding antagonists (1 µM AM 630 for CB_2_R or 1 µM YIL 781 for GHSR1a) or vehicle and stimulated with agonists (200 nM JWH-133 for CB_2_R or 200 nM ghrelin for GHSR1a) (15 min) before the addition of 0.5 μM forskolin or vehicle. Finally, reaction was stopped by addition of the Eu-cAMP tracer and the ULight-cAMP monoclonal antibody prepared in the “cAMP detection buffer” (PerkinElmer). All steps were performed at 25º. Homogeneous time-resolved fluorescence energy transfer (HTRF) measures were performed after 60 min incubation using the Lance Ultra cAMP kit (PerkinElmer, Waltham, MA, USA). Fluorescence at 665 nm was analyzed on a PHERAstar Flagship microplate reader equipped with an HTRF optical module (BMG Lab technologies, Offenburg, Germany).

### 4.9. MAPK Phosphorylation Assays

To determine MAP kinase 1/2 (ERK1/2) phosphorylation, striatal neurons were plated in transparent Deltalab 96-well plates and kept in the incubator for 15 days. 2 to 4 h before the experiment, the medium was replaced by serum starved medium. Next, the cells were pre-treated at 25 °C for 10 min with antagonists (1 µM AM 630 for CB_2_R or 1 µM YIL 781 for GHSR1a) or vehicle and stimulated for an additional 7 min with selective agonists (200 nM JWH-133 for CB_2_R or 200 nM ghrelin for GHSR1a). Then, neurons were washed twice with cold PBS before the addition of 30 µL/well “Ultra lysis buffer” -PerkinElmer- (20 min treatment). Afterwards, 10 µL of each supernatant were placed in white ProxiPlate 384-well plates and ERK1/2 phosphorylation was determined using an AlphaScreen^®^ SureFire^®^ kit (PerkinElmer), following the instructions of the supplier, and using an EnSpire^®^ Multimode Plate Reader (PerkinElmer, Waltham, MA, USA). The reference value (100%) was the value achieved in the absence of any treatment (basal). The ligands effect was given in percentage with respect to the basal value.

On the other hand, HEK-293T cells were cultured into 25 cm^2^ flasks and transfected with the cDNAs for CB_2_R (0.5 µg) and/or GHSR1a (1 µg). Two hours before initiating the experiment, cell-culture medium was exchanged to serum-starved DMEM medium. The cells were, subsequently, pre-treated at 25 °C for 10 min with antagonists (1 µM AM 630 for CB_2_R or 1 µM YIL 781 for GHSR1a) or vehicle and stimulated for an additional 7 min with selective agonists (200 nM JWH-133 for CB_2_R or 200 nM ghrelin for GHSR1a). Stimulation was ended by a rapid rinse with ice-cold PBS, and the cell lysis was performed by the addition of 250 µL of ice-cold lysis buffer. Cellular debris were removed by centrifugation at 13,000× *g* for 5 min at 4 °C, and protein was quantified by the bicinchoninic acid method using bovine serum albumin dilutions as standard. To determine the level of ERK1/2 phosphorylation, equivalent amounts of protein (10 μg) were separated by electrophoresis on a denaturing 10% SDS-polyacrylamide gel and transferred onto PVDF-FL membranes. Membranes were blocked with Odyssey blocking buffer (LI-COR Biosciences, Lincoln, NE, USA) for 60 min and probed with a mixture of a mouse anti-phospho-ERK1/2 antibody (1:2500, Sigma-Aldrich) and rabbit anti-ERK1/2 antibody (1:40,000, Sigma-Aldrich) for 2 h. The 42 and 44 kDa bands corresponding to ERK 1 and ERK 2 were visualized by the addition of a mixture of IRDye 800 (anti-mouse) antibody (1:10,000, Sigma-Aldrich) and IRDye 680 (anti-rabbit) antibody (1:10,000, Sigma-Aldrich) for 1 h and scanned by the Odyssey infrared scanner (LI-COR Biosciences). Band densities were quantified using the scanner software and exported to Microsoft Excel. The level of phosphorylated ERK 1/2 was normalized for differences in loading using the total ERK1/2 protein band intensities.

### 4.10. Intracellular Calcium Mobilization

HEK-293T cells were co-transfected with cDNAs for CB_2_R (0.5 µg) and/or GHSR1a (1 µg) in the presence of 1 μg cDNA for the calmodulin-based calcium GCaMP6 sensor. Forty-eight hours after transfection, cells were detached using Mg^2+^-free Locke’s buffer pH 7.4 (154 mM NaCl, 5.6 mM KCl, 3.6 mM NaHCO_3_, 2.3 mM CaCl_2_, 5.6 mM glucose and 5 mM HEPES) supplemented with 10 μM glycine. 1500 cells per well were plated in 96-well black, clear bottom, microtiter plates. Then, cells were incubated for 10 min with the CB_2_R and GHSR1a antagonists (1 µM AM 630 or 1 µM YIL 781), and subsequently stimulated with selective agonists (200 nM JWH-133 or 200 nM ghrelin). Upon excitation at 488 nm, real-time 515 nm fluorescence emission due to calcium-ion complexed GCaMP6 was recorded on the EnSpire^®^ Multimode Plate Reader (every 5 s, 100 flashes per well).

### 4.11. Proximity Ligation Assays (PLAs)

Physical interaction between CB_2_R and GHSR1a were detected using the Duolink in situ PLA detection Kit (OLink; Bioscience, Uppsala, Sweden) following the instructions of the supplier. Primary neurons were grown on glass coverslips, fixed in 4% paraformaldehyde for 15 min, washed with PBS containing 20 mM glycine to quench the aldehyde groups and permeabilized with the same buffer containing 0.05% Triton X-100 (20 min). Then, samples were extensively washed with PBS. After 1 h incubation at 37 °C with the blocking solution in a pre-heated humidity chamber, primary cultures were incubated overnight in the antibody diluent medium with a mixture of equal amounts of mouse anti-CB_2_R (1/100; sc-293188, Santa Cruz Technologies, Dallas, TX, USA) and rabbit anti-GHSR1a (1/100; ab95250, Abcam, Cambridge, United Kingdom) to detect CB_2_R–GHSR1a complexes. Neurons were processed using the PLA probes detecting primary antibodies (Duolink II PLA probe plus and Duolink II PLA probe minus) diluted in the antibody diluent solution (1:5). Ligation and amplification were done as indicated by the supplier. Samples were mounted using the mounting medium with Hoechst (1/100; Sigma-Aldrich) to stain nuclei. Samples were observed in a Leica SP2 confocal microscope (Leica Microsystems, Mannheim, Germany) equipped with an apochromatic 63× oil immersion objective (N.A. 1.4) and 405 and a 561 nm laser lines. For each field of view, a stack of two channels (one per staining) and four Z stacks with a step size of 1 μm were acquired. The number of neurons containing one or more red spots versus total cells (blue nucleus) was determined, and the unpaired Student’s t-test was used to compare the values (red dots/cell) obtained in the two groups.

## Data Availability

Data are available upon reasonable request to corresponding authors. A Western blot compilation file accompanies the submission and may be found via IJMS.

## Supplementary Materials

Supplementary materials can be found at https://www.mdpi.com/article/10.3390/ijms22168928/s1.
