# Supplementary material for: Identification of the Ghrelin and Cannabinoid CB2 Receptor Heteromer Functionality and Marked Upregulation in Striatal Neurons from Offspring of Mice under a High-Fat Diet
Source: Int J Mol Sci. 2021 Aug 19;22(16):8928. doi: 10.3390/ijms22168928 (PMC8396234; doi:10.3390/ijms22168928)

Supplementary material
Identification of the ghrelin and cannabinoid CB_2 receptor heteromer. Functionality and marked upregulation in striatal neurons of siblings of mice under a high-fat diet.

Running title. Ghrelin-cannabinoid receptor interactions

Jaume Lillo[1,2,#], Alejandro Lillo[3,#], David A. Zafra[3], Cristina Miralpeix[4], Rafael Rivas-Santisteban[1,2], Núria Casals[4,5], Gemma Navarro[1,3,6 ,#*] Rafael Franco[1,2,7, ,#*],

Affiliations:

[1] Centro de Investigación Biomédica en Red Enfermedades Neurodegenerativas (CiberNed), National Institute of Health Carlos iii. Valderrebollo, 5, 28031 Madrid, Spain.

[2] Departament de Bioquímica i Biomedicina Molecular, Universitat de Barcelona, 08028 Barcelona, Spain

[3] Department of Biochemistry and Physiology, Faculty of Pharmacy and Food Science, University of Barcelona, Barcelona, Spain

[4] Basic Sciences Department, Faculty of Medicine and Health Sciences, Universitat Internacional de Catalunya, Sant Cugat del Vallès, Spain

[5] Centro de Investigación Biomédica en Red de Fisiopatología de la Obesidad y la Nutrición (CIBEROBN), Instituto de Salud Carlos III, Madrid, Spain

[6] Institut de Neurociències de la Universitat de Barcelona (UBNeuro), Barcelona. Spain

[7] School of Chemistry. University of Barcelona, Barcelona, Spain.

# Equal contribution

***Corresponding authors:**

Rafael Franco (rfranco123@gmail.com; rfranco@ub.edu)
School of Biology. Universitat de Barcelona.
Diagonal 643. Prevosti Building.
Barcelona 08028. Spain
Tel +34 934021208

Gemma Navarro (g.navarro@ub.edu)
School of Pharmacy. Universitat de Barcelona
Joan XXIII, 29-31
Barcelona 08028. Spain
Tel +34 934034500

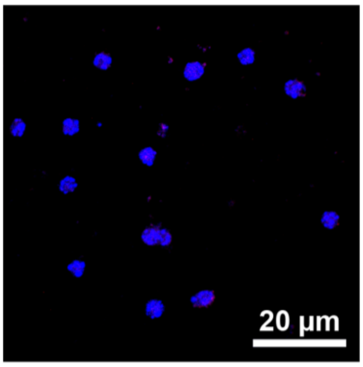

Supplementary Figure S1: Negative control of the *in situ* proximity ligation assay (PLA) in primary striatal neurons, undertaken by omitting one of the primary antibodies. Similar results were performed in samples from 5 animals coming from SD mothers and 5 from HFD mothers. Scale bar: 20 μm.

Western compilation:

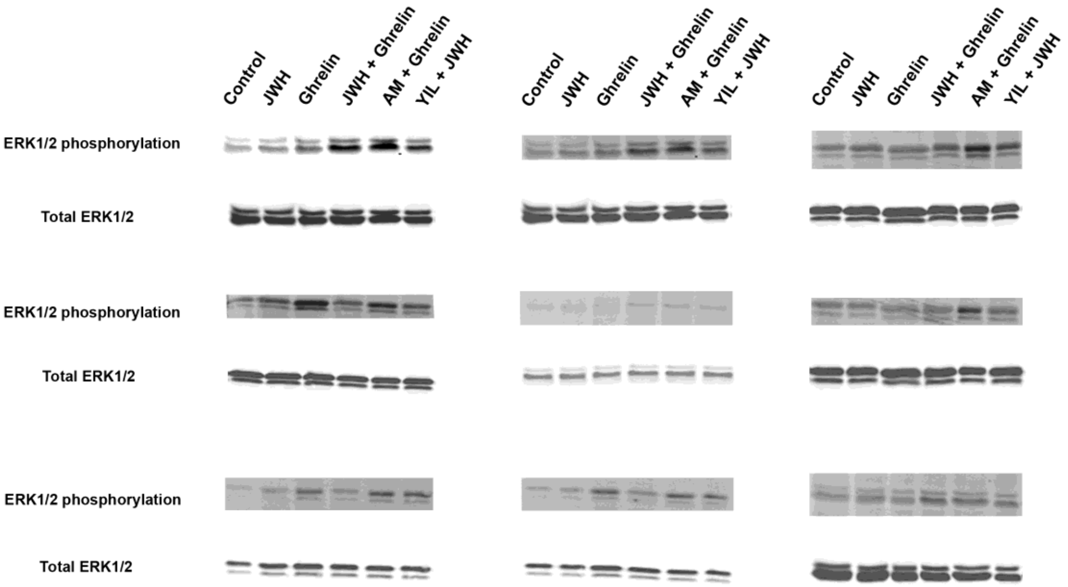

Supplement: Supplementary file 1 [file ijms-22-08928-s001.zip › ijms-1303008-supplementary.pdf]
